# Problematic substance use among patients in a Swedish outpatient psychiatry setting: staff and manager perceptions of digital options for increased intervention access

**DOI:** 10.1186/s13722-023-00421-x

**Published:** 2023-10-24

**Authors:** Elisabeth Petersén, Hanna Augustsson, Anne H. Berman

**Affiliations:** 1 Centre for Psychiatry Research, Department of Clinical Neuroscience, Karolinska Institute, Stockholm, Sweden; 2 Stockholm Health Care Services, Stockholm, Sweden; 3 Procome Research Group, Medical Management Centre, Department of Learning, Informatics, Management and Ethics, Karolinska Institute, Stockholm, Sweden; 4 Department of Psychology, Uppsala University, Uppsala, Sweden

**Keywords:** Substance use disorder, Comorbidity, Digital interventions, Staff perceptions

## Abstract

**Background:**

Approximately 50% of the patients who globally seek help in psychiatry have been assessed with problematic substance use or been diagnosed with substance use disorder (SUD). Given the high treatment gap for mental health care, in particular SUD, these individuals risk poorer treatment outcomes in psychiatry. Integrated treatment for psychiatric and SUD disorders has been proposed to reduce the treatment gap for SUD, but access to integrated treatment is low. Digital interventions addressing SUD in psychiatry could potentially make treatment available to patients who otherwise would not have access. In this study “digital interventions” comprise an umbrella term covering all kinds of interventions from minimal motivational app-based interventions to internet-based interventions with and without human guidance, up to remote sessions in telepsychiatry. This study aims to explore healthcare staff perceptions of referring patients to digital interventions for reducing problematic substance use, whether or not diagnosed as SUD, in the psychiatric outpatient setting.

**Method:**

The study was exploratory with a qualitative design. Data were collected in the Swedish outpatient psychiatry setting, via individual semi-structured interviews with managers, and focus groups with healthcare staff. An adapted form of phenomenological hermeneutical analysis was used to analyze the data.

**Results:**

Three themes emerged from the analysis. The first theme was Encountering obstacles on the path to future implementation of digital interventions, with sub-themes: *Lacking resources* and *Feeling concerned about technical solutions*. The second theme was Searching for ways forward to achieve increased access to care, with sub-themes: *Blended care could facilitate integrated care* and *Addressing variations in patients’ technical skills*. The third theme was Taking steps towards the future, with sub-themes: *Wanting to know more about digital interventions* and *Formulating a vision for the future*.

**Conclusions:**

The study reveals a concern that implementing digital interventions in psychiatry will create additional work or be technically challenging. The staff see significant advantages from the patient perspective, but they feel that they themselves need training in implementing digital interventions. In order to establish constructive implementation of digital interventions for SUD in psychiatry, staff attitudes and concerns need to be considered and addressed. This study was conducted within the Swedish healthcare system and the findings may not generalize to other countries with differing healthcare systems.

**Supplementary Information:**

The online version contains supplementary material available at 10.1186/s13722-023-00421-x.

## Background

A large proportion of people are living with mental illness in the world [1] and in 2017, more than one in ten people expressed that they suffered from mental illness [2]. Comorbidity is common in this patient group [3] and it is estimated that about 50% of patients who seek help in psychiatry at some point in life have had or will be diagnosed with substance use disorder (SUD; 4, 5). A recent survey of comorbidity in Sweden showed that among individuals treated for SUD, 52% of the men and 64% of the women had also been treated for a psychiatric diagnosis [6]. However, a large treatment gap obtains between those who need care and those who actually receive it [7]. An estimate of median treatment gap for patients living with alcohol dependence in Europe was at an alarming 92.4% in 2004 [8]; the figures for illicit drug use were at similar levels, without significant improvement over the past two decades [9]. The treatment gap for SUD is significantly larger than for mental disorders such as schizophrenia, major depression, bipolar disorder or generalized anxiety [8].

Individuals with mental illness and problematic substance use comorbidity have a higher risk of family problems, isolation, financial problems, as well as school- or work-related difficulties [10–13]. Several different mental illnesses, such as depression [14], bipolar disorder [15], anxiety [16] and an increased risk of suicide [17, 18], are linked to long-term alcohol consumption. A systematic review concluded that treatment of mental illness or SUD alone did not have an effect on both conditions [19]. Individuals with comorbidity would thus need to receive treatment and support for both conditions at the same time [13], but they often have more complex needs [11] and treating both conditions in parallel can be challenging [20]. When the two conditions are treated at separate clinics there is a risk that the patient “falls between the cracks” [21]. Ideally, integrated treatment for mental illness and SUD could be delivered at the same clinic, and could be a way to retain patients with comorbidity in standard psychiatric care. This would require training and supervision to introduce and maintain quality care [22]. A review from 2019 concluded that *digital interventions* for SUD in psychiatry could resolve the multiple barriers to integrated treatment; however, no evidence was found of published trials on digital interventions for substance use within the mental healthcare setting [23].

Research on integrated treatment among community dwellers outside the mental healthcare setting has shown some promising results. An early study showed that a digital integrated intervention, targeting both SUD and depression, was at least equivalent to face-to-face treatment [24]. A systematic review from 2019 based on 28 studies among community dwellers concluded that digital integrated interventions delivered in combination with therapist support yielded better effects than digital interventions alone [25]. A systematic review and meta-analysis from 2022 in community-based populations, based on a total of 8 studies with 6 that qualified for meta-analysis, found that integrated treatment with digital interventions can have a positive effect on patients with depressive symptoms after follow-up at 3 months and for alcohol consumption after 6 months [26]. However, an additional systematic review and narrative synthesis from 2022, based on 5 studies, concluded that there is still no clear evidence that integrated treatment via digital interventions for patients with comorbidity in community dwelling populations has sufficient effectiveness [27].

Returning to the question of offering digital interventions for SUD within the psychiatric context, this could increase access to treatment for patients that have historically had lower access to SUD treatment, such as young people, women and at-risk users [28]. In this study, “digital interventions” comprise an umbrella term covering all kinds of interventions from minimal motivational app-based interventions to internet-based interventions with and without human guidance, up to remote sessions in telepsychiatry. Studies have shown that older adults over 60 years are less likely to use such online services, but with training and user-friendly online services, adapted for the elderly, there is a chance of reducing the risk of digital exclusion, though it is still important to continue to offer usual care as an alternative [29]. Preliminary results also show that patients who did not seek help via traditional face-to-face treatment were attracted by the possibility of signing up for digital interventions [30]. Blended care, meaning a combination of face-to-face therapy and digital interventions, is another potential way to reach patients [31] that can reduce the risk of early dropout and increase patients' treatment adherence [32].

If patients can be engaged in digital SUD treatment within psychiatry, it is important to note that the evidence base for digital interventions for SUD is increasingly strong. During the past decade, research on digital interventions in the form of web-based interventions for problematic alcohol consumption with guidance has increased and shown small but significant positive effects [33]. Digital interventions have significantly better effects in reducing alcohol consumption in comparison with control groups [33, 34]; comparisons with waitlist participants in RCT:s yielded significantly better treatment results compared to other types of control groups such as no intervention, or brief/minimal intervention [33]. In a comparison between high versus low intensity digital interventions both groups showed reduced alcohol consumption over short- and long-term follow-ups up to 24 months, with no significant difference between high and low intensity treatments [35]. In addition, a network meta-analysis showed that digital interventions for alcohol use disorders are effective alternatives, further concluding that implementation of digital interventions in clinical praxis can mitigate several barriers such as economics, geography and lack of time [36]. In sum, lowering the threshold for access to evidence-based substance use treatment within psychiatry seems to be an urgent priority and an exploration of barriers and facilitators for introducing digital interventions in the mental healthcare context is thus warranted.

In Sweden, all residents have access to healthcare through their local region for a low co-payment fee with a maximum yearly fee of 110 €. About 85% of residents state that they have access to the healthcare they need, with some regional variations [37]. A national survey of attitudes towards digital healthcare in 2022 showed that 45% of residents in Sweden have a positive attitude towards digital care, and that women are more positive (47%) than men (42%) [37]. Since 2007, digital interventions for psychiatric disorders have been offered nationally in Sweden via Internet Psychiatry, a designated clinic targeting psychiatric care [38]. A robust body of research from this clinic and others worldwide has additionally shown the effectiveness of internet-based psychological interventions, largely based on cognitive-behavioral therapy, for common mental disorders such as depression and anxiety, in comparison to face-to-face treatment [39, 40]. When a national platform for digital interventions in all healthcare contexts was launched in 2015, the digital intervention menu in primary, somatic and psychiatric contexts began to vastly increase. Since 2018, data on internet-based psychological treatment offered in primary, somatic or psychiatric contexts have been collated in a national registry, showing that 18 778 patients have been treated with digital psychological interventions and over 70% began treatment within one month from first request for care [41, 42]. Also, since 2018, internet-based treatment for substance use has been available nationally through self-referral via a web portal sited in addiction care; patients within psychiatry can access this care via self-referral or formal referral from psychiatric treatment providers [38]. However, internet-based treatment within psychiatry has still not targeted substance use [43].

The research literature has not addressed how digital interventions could be applied to address the specific needs of patients in psychiatry with comorbid substance use [44]. Our own research in this area began with a survey investigating the practices of psychiatry staff in their encounters with patients in psychiatry that might have comorbid substance use. We found that management and staff attitudes towards digital interventions could be a key issue that might illuminate barriers and facilitators to the challenges of treating comorbidity in the psychiatric context [45]. This study thus aims to explore the perceptions of healthcare staff regarding the possibility of referring patients to digital interventions for reducing problematic substance use, as well as diagnosed SUD, in the psychiatric outpatient setting.

## Methods

### Study design

This was an exploratory study with a qualitative design. Data were collected in outpatient psychiatry via individual semi-structured interviews with managers and focus groups with healthcare staff. An adapted form of phenomenological hermeneutic analysis was used to analyze the interviews [46].

### Participants and procedure

The managers were identified from a group of respondents to a previous national survey study [45]. In the survey, over 60% of participants had over 10 years of experience working in psychiatry, over 15% had between 6 and 10 years of experience and over 20% had under 5 years of experience [45]. Respondents were not asked to specify their work experience in the interviews reported here. In total, 10 managers were included in the individual interview study, conducted in 2014–2015. Three managers agreed to facilitate staff participation in focus groups, which were conducted in 2015 with a total of 18 participants. The majority (n = 20/28) of those interviewed individually and in focus groups, were women. The inclusion criteria for managers were employment as head of the clinic and the inclusion criteria for healthcare staff participating in focus groups were employment at the clinic and working clinically with patients. The individual interviews were conducted at different types of clinics, specializing in different disorders, including psychosis, borderline disorders, ADHD, general psychiatry and affective disorders. Focus group interviews were conducted at a general psychiatric clinic, an ADHD clinic and a psychosis clinic. All clinics were located in the Stockholm region (Table 1).

**Table 1:** Presentation of participants and their occupations

Occupation	Individual interviews with managers	Focus group interviews with staff
Participants		
Nurse/specialist nurse	5	6
Psychologist	4	2
Physician	–	4
Social worker	1	2
Mental health worker	–	1
Psychology student	–	2
Psychotherapist	–	1

Participants gave their informed consent for participation in the study. Ethical review by the Stockholm Regional Ethical Review Board yielded a consultative statement stating that no ethical approval was needed (Ref. No. 2012/1695-31/5).

All individual interviews and focus groups were semi-structured and conducted by two interviewers, one who led the interview and one who observed and, if appropriate, asked follow-up questions. The individual interviews lasted 36–54 min and focus groups lasted 65–80 min. To stimulate participants’ thinking about digital interventions within the psychiatric context, in both the interviews and focus groups, they were presented with a possible stepped care model for digital treatment of problematic alcohol and drug use, building on then ongoing research [47–52]. The interviews were conducted, transcribed and analyzed in Swedish. The quotes in the text and the interview guides were translated into English after the analysis. All translated text was reviewed and edited by author AHB, whose native language is English. See Supplementary information (Additional files 1, 2) for interview guides. No compensation was offered except for buns to accompany coffee/tea in the focus groups.

### Data analysis

The analysis in this study is based on narrative interviews, where the data collected create an image of the phenomenon explored; this image is interpreted in turn, based on the reality described in the interviews [46]. The digitally recorded interviews were transcribed via a professional transcription service. All interviews were repeatedly listened to and read through before initiating formal analysis, here termed *structural analysis* of the interviews. The data analysis was adapted from the phenomenological hermeneutic process [46], which meant that analysis began with a naive reading of the interviews, documented in writing as the *naive understanding* (Additional file 3) and representing author EP's perception of the material collected. Authors AHB and an additional colleague contributed to the continued analysis by listening, reflecting and discussing the data material. In the structural analysis, meaningful units were picked out and condensed into shorter sentences. These abstractions were then printed on paper, individually cut out and then placed on a large table where the notes were then grouped according to similarity with each other. This process was repeated several times before the groupings led to the final themes and sub-themes [46]. To increase the validity of the study, these were then compared with the original naive understanding [45]. The quotes are presented with numbers: XX:XX, where the first number indicates an individual interview with a manager if marked with 1–10, or a focus group, marked with 11–13. The second number shows the meaning unit identifier.

## Results

Based on the structural analysis, three themes emerged that shed light on psychiatry staff perceptions of future referrals to digital interventions to reduce problematic substance use or SUD in the psychiatric outpatient clinic. Table 2 shows three themes with two sub-themes each. The detailed flow of the structural analysis from meaningful units to themes is shown in Table 3.

**Table 2:** Overview of Themes and Sub-themes

Theme	Sub-theme 1	Sub-theme 2
Encountering obstacles on the path to future implementation of digital interventions	Lacking resources	Feeling concerned about technical solutions
Searching for ways forward to achieve increased access to care	Blended care could facilitate integrated care for comorbidity	Addressing variations in patients’ technical skills
Taking steps towards the future	Wanting to know more about digital interventions	Formulating a vision for the future

**Table 3:** Example of the structural analysis from meaning units to themes

Meaning unit	Condensation	Abstraction	Sub-theme	Theme
9:49 Yes, but I think…that I understand that they are down on their knees, we are too, but I also think that, to a certain extent, that we are on our knees because [the substance abuse] is such a problem	I understand that they are down on their knees, we are too, but to a certain extent we are on our knees because [the abuse] s such a problem	Down on one’s knees, partly because of this kind of problem [abuse]	Lacking resources	Encountering obstacles on the path to future implementation of digital interventions
12:64 And for me, it's that I'm not that knowledgeable when it comes to computers at all, so I look at [name of colleague], and technology and so on, and it's not out of disinterest, but I think it's more about what happens when I start with this, that it is important for me then in that case or someone else who feels this uncertainty to get, that I as a staff member then get good, clear information about how this should be able to work	I'm not very knowledgeable when it comes to computers at all and it's not out of disinterest but I think it's more about what happens when I start with this, that it is important to me then in that case or someone else who feels this uncertainty to get, that I as a staff member then get good, clear information about how this should work	Not computer literate but not disinterested–feels insecure–important with information	Feeling concerned about technical solutions	Encountering obstacles on the path to future implementation of digital interventions
7:42 If I were to imagine that I would work with addiction care, then I would think that this is great because then this is something that the patient can work with between the times you are seen. You can look at the pattern they have, you can look at how they handle risk situations or what triggers increased drug use, etc., so then I would think it is great because all types of problem solving or treatment must include the patient	If I were to work with addiction care, then I would think that this is great because then this is something that the patient can work with between the times you are seen. You can look at the pattern they have, then I would think that it is great because all types of problem solving or treatment must include the patient	Great—the patient can work between the times we meet. Great for problem solving to see the pattern and include the patient	Blended care could facilitate integrated care	Searching for ways forward to achieve increased access to care
13:75 Yes, partly they are technically proficient, they have that opportunity and they are already in the app world and all this and understand, they are people who thrive when such things happen and yes, if the others would then I, so they would get super anxious from it and just feel that no, now I'm even worse than I am and now I cannot handle this either and like, yes	Technically proficient, they have that opportunity and they are already in the app world while others would get super anxious from it and just feel that no, now I am even worse than I am and now I cannot do this either	Technically proficient people who are already in the app world are more comfortable than others who can get super anxious	Addressing variations in patient´s technical skills	Searching for ways forward to achieve increased access to care
11:88 Yes and so it is now, that we have a lot of applications that, this is good, this is good, but no one has really gotten into it like that, but if you ensure the quality of these, you can still feel that we trust that you have done a good job and that you have developed this properly and then we can refer to it, it would feel good, that it was through that, that it was picked up	We have a lot of applications that are good, but no one has really gotten into it so, if you ensure the quality of these, you can still feel that we trust that you have done a good job and you have developed this properly and then can we refer to it, it would feel good	Many good applications but no quality assured that we can refer to	Wanting to know more about digital interventions	Taking steps towards the future
5:56 It’s about that I want, hey, I actually want to be part of the development	I want to be part of the development	Wants to be part of the development	Formulating a vision for the future	Taking steps towards the future

### Encountering obstacles on the path to future implementation of digital interventions

The first theme illustrated staff perceptions of the obstacles to introducing digital interventions and showed two sub-themes: (1) Lacking resources and (2) Feeling concerned about technical solutions. The first sub-theme, *Lacking resources*, meant feeling insufficient. This was visualized as experiencing stress at work that was difficult to handle, and also wishing to work in a more structured way but not having the opportunity to adapt working tasks under the prevailing circumstances. The respondents felt that implementing a digital intervention would mean yet another task to keep track of in their already congested workdays.

*‘‘ Yes, but I think…that I understand that they are down on their knees, we are too, but I also think that, to a certain extent, that we are on our knees because it is such a problem [SUD]”. 9:49*.

However, respondents also described feeling hope for the future. This was visualized by staff expressing that they were feeling hopeful about being able to change their working methods. Being able to change today's way of working so that resources would be redistributed could contribute to implementation of digital interventions, which might be a time-consuming task but in the long run be positive in their work.

“*There are many parts you want to improve, as now we have talked a lot about this, with those who have children and it is difficult to take everything at once, but this particular thing with SUD, I think we think a bit like we have this routine but of course, if you had the time and energy and opportunity, you would probably improve it, that’s my opinion.” 1:23.*

The second sub-theme, *Feeling concerned about technical solutions*, meant adapting ways to use new systems in one’s clinical context and motivating the staff to use new technical solutions, seeing them as an aid rather than an obstacle. This was visualized by the staff expressing concern about learning and using new systems.

*‘‘And for me, it's that I'm not that knowledgeable when it comes to computers at all, so I look at [name of colleague], and technology and so on, and it's not out of disinterest, but I think it's more about what happens when I start with this, that it is important for me then in that case or someone else who feels this uncertainty to get, that I as a staff member then get good, clear information about how this should be able to work.” 12:64.*

Respondents also expressed concern about not meeting the patient in person. They were afraid that a technical intervention would not be as good as an in-person appointment and that the patients would not feel seen or validated in their illness. Replacing treatment providers’ human contact with the computer screen at home would partly mean that the staff would be competing with technical solutions but also that it could increase the patients’ feelings of loneliness.

*“I'm a little skeptical that there is not a human being who makes contact—because is that what is offered? And many of these patients are already lonely, and this is going to be different, yes, but that's it. Society is moving more and more towards digitalization”. 13:57*.

### Searching for ways forward to achieve increased access to care

The second theme illustrated staff perceptions of the role of digital interventions to achieve increased access to care and included the following sub-themes: (1) Blended care could facilitate integrated care (2) Addressing variations in patient´s technical skills. The first sub-theme, *Blended care could facilitate integrated care*, illuminated the possibility of patients having an opportunity, between in-person appointments, to choose between various technical solutions, such as an app for a mobile phone or internet-based texts to support changing addictive behaviors. It meant perceiving how digital interventions could be a support in patients’ everyday life. This was visualized by the staff expressing that they had a vision of how a digital intervention could create an opportunity for patients to work more actively with their problems and increase their participation in their treatment.

*‘‘If I were to imagine that I would work with addiction care, then I would think that this is great because then this is something that the patient can work with between the times you are seen. You can look at the pattern they have, you can look at how they handle risk situations or what triggers increased drug use, etc., so then I would think it is great because all types of problem solving or treatment must include patients”. 7:42.*

Respondents also described how blended care could facilitate adaptation of treatment to patient needs. Information about a digital opportunity to supplement treatment could facilitate the change process, while it would be important to ensure that there is another alternative if the patient would not feel comfortable using digital interventions. For example, the digital intervention could function as a complement to regular care when patients also felt a need for human contact.

*‘‘Yes, making demands and not being able to cope… it is important that you always give information that even if this does not work, there is another way and that it is almost never impossible; rather, it is just as important that, if it is the case that you cannot do it, then there should be some kind of counterbalance that weighs up the feeling of failure”. 13:70.*

The second sub-theme, *Addressing variations in patient´s technical skills*, meant both safeguarding the skills that today’s patients have, but also seeing what future patients’ needs might be. This was visualized by the respondents recognizing that much of modern life is becoming increasingly digitalized. The staff noted that older generations did not have access to the internet or learn to use smartphones. In contrast, younger people have grown up with technology and are used to working with computers and telephones from an early age, particularly given that a large proportion of social contacts for young people take place today via social media.

*‘‘ But then there is the fact that if they are younger, they are also used to social contacts via the internet, they are more involved in their social activities there than those who are a little older, so that in the future I think you can then book an appointment online and have one communication via chat. I think that is possible”. 12:76.*

*Addressing variations in patient´s technical skills* also meant seeing an opportunity to lower the threshold for seeking help and working towards the possibility that all patients could be offered care in an appropriate manner. For some patients it could mean a physical visit to a clinic, while for other patients it could be completely digitized treatment contacts. By offering digital interventions, patients who do not want to seek in-person care might dare to take the step and ask for help. This could reduce feelings of guilt and shame and hopefully also reduce feelings of stigma that many patients experience in contact with psychiatry or addiction care.

*‘‘ And that's what's so sad, so that's the question, I can imagine that a modern, technical, solution would do, open up so that you reach more, so just like internet psychiatry makes them come, people who do would not seek out psychiatry so maybe this can open things up and reach people who would not seek out the addiction clinic so I think, yes, hurry it up, absolutely”. 4:51*.

### Taking steps towards the future

The third theme visualized the staff's thoughts about daring to take steps towards future care that could offer a greater digital range of treatments. This included the following subthemes: (1) Wanting to know more about digital interventions; and (2) Formulating a vision for the future. The first sub-theme, *Wanting to know more about digital interventions*, meant trying to update old knowledge and being interested in searching for a new path. This was visualized by respondents’ showing interest in finding new ways of working and being open to identifying advantages of using and implementing new systems such as digital interventions in their daily work to continue helping the patients find a way to recovery.

*“So if we feel that, yeah, this is really something that is useful for us, then it will work, that’s what I would like to say.” 8:66*.

Respondents felt that it was important to stay up to date on what treatments were available in order to be able to identify the needs of the individual patient. It was deemed important to focus on the users in the development of digital interventions—both patients and staff—in order to be able to develop programs that are user-friendly and to offer staff training in the programs as well as easily accessible support for all users. By understanding the digital interventions, treatment providers could also advocate for using them.

*‘‘Yes and so it is now, that we have a lot of applications that… [are] good…but if you [clinical researchers] ensure the quality of these, you can still feel that we trust that you have done a good job and that you have developed this properly and then we can refer to it, it would feel good…that it was picked up.”. 11:88*.

The second sub-theme, *Formulating a vision for the future*, meant having a vision of being part of digital treatment development, and wanting to contribute to the healthcare of the future. This was shown by the respondents describing how expanded services would provide greater opportunities and that this was in line with in the rest of society becoming more digitally based. Being innovative and learning to work with new technology was seen as a positive part of the work.

*‘‘…I actually want to be part of development, we all just want the patient to receive the best care available and if there is research that may be able to drive development, so hey, of course we should be involved there”. 5:56*.

Respondents also recognized the benefits of being able to offer digital interventions for SUD in the future, to complement existing psychiatric treatment. Respondents described how a digital intervention could function as a bridge to integrated treatment in psychiatric healthcare. Patients who reduced their substance use could gain a mental and physical advantage by increasing their overall wellbeing (see e.g., 53).

*‘‘ And exactly, in the best of worlds, then perhaps such a system could bridge the gap that I still believe exists between addiction care and, for example, the borderline unit and other psychiatric clinics”. 2:56.*

### Comprehensive understanding and discussion

The comprehensive understanding of the results is the interpreted whole of the present study. Managers’ and staff perceptions of future referral to digital interventions for addressing SUD in the psychiatric outpatient setting were illuminated based on three themes: Encountering obstacles on the path to future implementation of digital interventions, Searching for ways forward to achieve increased access to care and Taking steps towards the future. The overall analysis shows that staff expressed a desire to have access to quality technical healthcare solutions that could facilitate their work and improve the patient's care. At the same time, there was a great deal of concern about not being able to cope with the new technical solutions and being saddled with another burdensome task in an already stressed work environment. This can be interpreted as a desire to have access to the technical aids but at the same time not necessarily to be involved in implementing them.

**Fig. 1:**
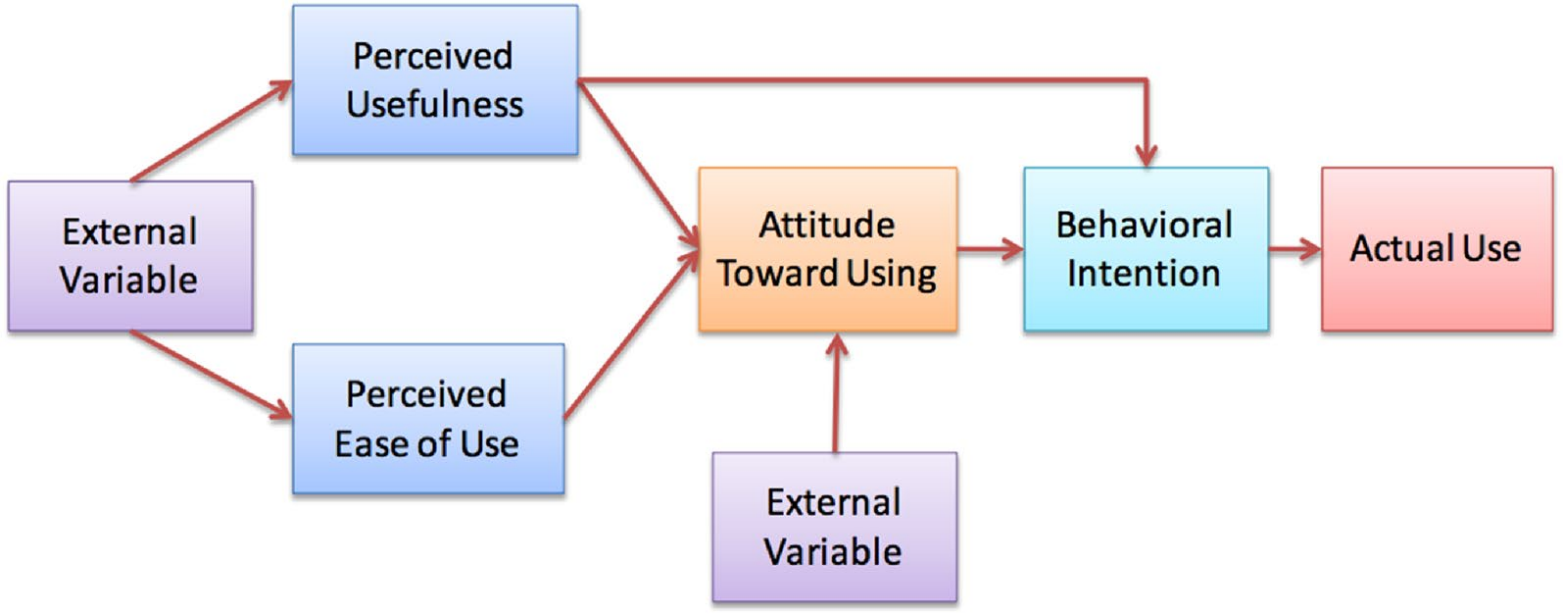
Technology acceptance model. Reproduced with authors’ permission

The findings imply that the respondents are interested in new solutions that can facilitate the treatment of patients but that they currently lack sufficient resources to be able to implement such technical solutions. There is also a concern that the technical solution will not facilitate the work but rather increase the workload, for example through double documentation due to parallel systems. Our findings can be interpreted within the framework of the Technology Acceptance Model (TAM; 54) which examines users' acceptance of an information system (see Fig. 1). According to the TAM, perceived usefulness and perceived ease of use lead to the user's attitude towards using the technology. In the next step, attitudes towards using the technology, together with perceived usefulness, affect the behavioral intention to use the technology. Other external factors can influence attitudes strongly [54], where examples might be patient openness to using technology, or the healthcare organization’s support for implemention of new technology.

The sub-theme Feeling concerned about technical solutions meant that staff could see the benefits of digital interventions, provided that all the technical obstacles were removed. This can be interpreted in relation to the second factor in Technology Acceptance Model, which is about perceived ease of use, in turn meaning that such use should be able to take place without much effort [54]. On the other hand, external variables such as training prior to use, could positively affect the perceived benefit and the perceived user-friendliness [54]. Offering psychiatry staff training concerning the digital interventions could thus help overcome the barrier of uncertainty regarding digital interventions and facilitate implementation of new ways of offering integrated treatment.

Patient participation is stated to be one of the most important factors in being able to ensure that the patient receives safe, quality care [56]. This was shown in the current study by the staff believing that digital interventions would increase the patient’s participation in their care. If blended care were introduced to a greater extent than today, patient participation in SUD treatment might increase. Blended care gives patients the opportunity to receive an even more individually tailored treatment as the technology to some extent can replace previous face-to-face contact [31]. A combination of digital and human contact suits some individuals better than others, and digital interventions that also include some form of human support have been found to be somewhat more effective than those that are solely self-guided or fully automated [57]. In order for patients and staff to benefit from blended care, it is important to identify which patients benefit from the blended model [31]. This in turn implies a need for learning from patients' participation when designing interventions, as well as a need for inter-professional learning [58, 59]. The role of the digital intervention is thus not only to complement in-person and/or enable blended care, but also to offer the patient direct access, whether or not psychiatry staff have training in SUD themselves. Increased training of staff in psychiatry could, nonetheless, facilitate offering patients individualized options for addressing their substance use, via in-person care within psychiatry, via blended care with support from psychiatry staff, or via digital interventions accessed independently by the patient, with minimal support from psychiatry staff. An additional path for offering digital interventions to patients in psychiatry could be collaboration with the initially mentioned e-support unit within addiction care that offers digital interventions for SUD nationally in Sweden.This unit has experienced continually increasing patient numbers, particularly self-referrals, and an expanded range of treatments, suggesting that patients are eager to independently access digital interventions for SUD [60].

Finally, well-designed and implemented digital interventions can contribute increased patient-centered and integrated care while increasing accessibility [61]. Initial research in another healthcare context, namely primary care, suggests that digital interventions could be a way to improve treatment of primary care patients with SUD [62, 63]. Digital interventions could contribute to enabling individuals to minimize their alcohol consumption [64] and also provide greater access to care for more patients [65, 66], in addition to increasing convenience for patients and reducing their isolation and sense of stigma [67]. Similar benefits could occur within the psychiatric context, if the barriers to introducing digital interventions for SUD could be overcome.

### Strengths and limitations

Study strengths include innovative exploration of possible barriers and facilitators in introducing digital interventions for SUD within the psychiatric context. Another strength is that the study population comprises several different staff categories, thus providing a comprehensive picture of the staff attitudes and perceptions. Study limitations are a small study population, and limited documentation of demographic characteristics, including age, to reduce the risk of revealing the anonymity of the study population.

## Conclusion

There has been an increasing interest in implementing digital interventions in healthcare. However, this study demonstrates a concern that implementing digital interventions for SUD in psychiatry will create extra work and be technically difficult. For the patient’s sake, the staff see great benefits, but staff opportunities for training in digital interventions need to be expanded. Currently, support exists among staff for using digital interventions for SUD in psychiatry if the benefits for patients are clear, but implementation would require staff attitudes and resistance to be considered and adequately addressed. Once implementation barriers have been reduced, future research would also need to explore what kinds of digital interventions might fit different configurations of psychiatric comorbidity. This study was conducted within the Swedish healthcare system and the findings may not generalize to other regions that have different healthcare systems.

### Supplementary Information

**Additional file 1.** Interview guide: Digital interventions with psychiatry clinic managers (individual interviews).

**Additional file 2.** Digital Interventions, psychiatry: Interview guide for focus groups.

**Additional file 3.** Naïve understanding.

## Data Availability

The datasets used and/or analyzed during the current study are available from the corresponding author on reasonable request.
