# Supplementary material for: Problematic substance use among patients in a Swedish outpatient psychiatry setting: staff and manager perceptions of digital options for increased intervention access
Source: Addict Sci Clin Pract. 2023 Oct 24;18:65. doi: 10.1186/s13722-023-00421-x (PMC10594773; doi:10.1186/s13722-023-00421-x)
Supplement: Supplementary file 3 — Additional file 3. Naïve understanding. [file 13722_2023_421_MOESM3_ESM.docx]

**Naïve understanding**

Future ideas about implementing digital interventions were seen as something that was exciting and interesting but at the same time a bit difficult and sometimes perceived as scary. A fear of being replaced by a machine was mixed with a fear that the patient would not complete their treatment when they did not have in-person visits with the treatment provider. Overall, respondents could be perceived as positive to the introduction of digital interventions; however, there was also a desire to be able to select the parts of the treatment that suited the individual patient best and then use digital interventions in those parts. In offering digital interventions, healthcare was perceived as continually being developed, and keeping up with developments taking place in the rest of society, which also included supporting young people and other persons more accustomed to the internet. However, it was considered a problem that the staff did not always have sufficient computer knowledge for supporting patients using digital interventions; nonetheless, this obstacle could be overcome with training and setting aside time. Being able to offer digital screening and digital interventions was seen as an opportunity to bridge the gap between psychiatry and addiction care.
